# A rare case of ocular and testicular T-cell lymphoma in a hermaphrodite koi carp (*Cyprinus carpio* Linnaeus 1758): clinical, histopathological, and immunohistochemical study

**DOI:** 10.1186/s12917-023-03621-4

**Published:** 2023-04-27

**Authors:** Hooman Rahmati-Holasoo, Sara Shokrpoor, Amin Marandi, Niyousha Torjani, Hosseinali Ebrahimzadeh Mousavi

**Affiliations:** 1 Department of Aquatic Animal Health, Faculty of Veterinary Medicine, University of Tehran, Tehran, Iran; 2 Centre of Excellence for Warm Water Fish Health and Disease, Shahid Chamran University of Ahvaz, Ahvaz, Iran; 3 Department of Pathology, Faculty of Veterinary Medicine, University of Tehran, Tehran, Iran; 4 Faculty of Veterinary Medicine, University of Tehran, Tehran, Iran

**Keywords:** Anisokaryosis, CD3, Enucleation, Exophthalmia, Immunohistochemistry, Lymphoma, Metastasis, Ocular mass

## Abstract

**Background:**

Lymphatic neoplasia may occur in various types, such as lymphoma, lymphosarcoma, lympholeukemia, and plasmacytoid leukemia. Lymphoma, defined as a malignant tumour of lymphoid tissue, has been found in a number of fish families including Esocidae and Salmonidae. However, the occurrence of lymphoma is rare in those belonging to the Cyprinidae. A final diagnosis of ocular and testicular T-cell lymphoma in the present study was based on the clinical signs, morphology, and texture of the tumour masses in the macroscopic and microscopic examinations. In addition, histopathological and immunohistochemical findings corresponded to T-cell lymphoma characteristics.

**Case presentation:**

A 2-year-old hermaphrodite koi carp (*Cyprinus carpio* Linnaeus 1758) with a large ocular mass and severe exophthalmia in the right eye was referred to the Ornamental Fish Clinic in October 2020. Under anesthesia, enucleation was performed. 57 days after enucleation of the right eye, exophthalmia in the left eye was discovered. 221 days after surgery, the fish was discovered to be dead. At necropsy, a large soft tissue mass attached to the left testis was discovered. There were also small whitish nodules on the surface of the liver. Histopathology revealed a hypercellular ocular mass with scant connective tissue. The sections also revealed multifocal hemorrhages, round to ovoid neoplastic cells, mild-to-moderate anisokaryosis and anisocytosis, and mitotic figures. Basophilic neoplastic cells were found in blood vessels within the testicular mass, raising the possibility of systemic spread. The liver showed microscopic metastasis with morphologic similarities to the ocular and testicular tumors. The neoplastic cells infiltrating the left and right eyes as well as the testicular mass were immunohistochemically positive for CD3 but negative for CD20. The masses were diagnosed as T-cell lymphoma based on histopathological and immunohistochemical findings.

**Conclusions:**

This case report provides the first evidence of clinical, histopathological, morphological, and immunohistochemical findings of an ocular and testicular T-cell lymphoma in a hermaphrodite koi carp (*Cyprinus carpio*) in Iran.

## Background

The aquaculture industry’s most important sectors are food and ornamental fish cultivation [1]. Although ornamental fish culture is a minor component of the global fish trade [2], it has evolved into one of the most important aspects of aquaculture [3–7], and it is recognized as one of the most profitable industries in many countries around the world [1, 2]. Cyprinids are one of the world’s most well-known and largest families of East Asian freshwater fish [8]. The koi carp (*Cyprinus carpio* Linnaeus 1758) as a freshwater ornamental fish is a coloured variety of common carp (*C. carpio*) that originated in Japan and has been bred in Iran since 2002 [9–11].

Neoplasia can occur in both lower and higher vertebrates [12]. Fish neoplasms are classified according to the mammalian tumour classification system [13, 14]. Lymphatic neoplasia may occur in various types, such as lymphoma, lymphosarcoma, lympholeukemia, and plasmacytoid leukemia [15]. According to veterinary literature, lymphomas are common in dogs, cats, and pigs, but comparatively rare in horses and other domestic species [16–19]. Lymphoma, defined as a malignant tumour of lymphoid tissue [18], has been found in a number of fish species including northern pike (*Esox lucius*) [20, 21], Japanese medaka (*Oryzias latipes*) [22, 23], coho salmon (*Oncorhynchus kisutch*) [24], black bullhead (*Ameiurus melas*) [25], rainbow trout (*Oncorhynchus mykiss*) [26], flower horn (hybrid cichlid) [27], gold crossback arowana (*Scleropages formosus*) [28], Atlantic stingray (*Hypanus sabinus*) [29] and captive white catfish (*Ameiurus catus* Linnaeus) [30].

In contrast to the epizootics of lymphoma in Esocidae [21] and Salmonidae [24], the occurrence of lymphoma is rare in those belonging to the Cyprinidae, and the current study serves to present the clinical, histological, and immunohistochemical characteristics of the first T-cell lymphoma from a hermaphrodite ornamental koi carp (*C. carpio*) in Iran.

## Case presentation

**Fig. 1:**
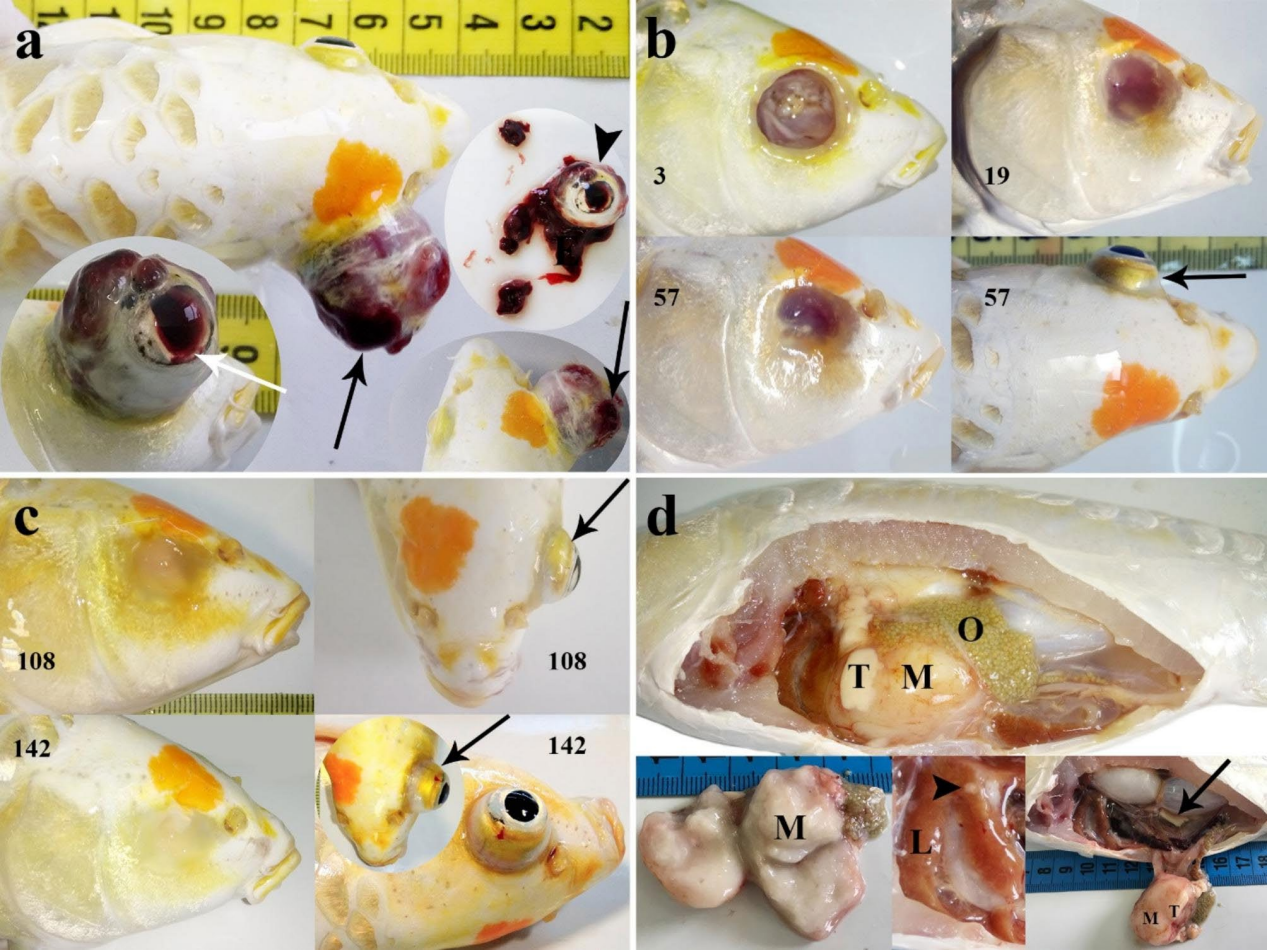
Clinical and necropsy images of a hermaphrodite koi carp (*Cyprinus carpio*). **(a)** Dorsal and lateral view of severe exophthalmia due to large and hemorrhagic mass (black arrows) in the right eye. Also, hemorrhage in the eye is seen (white arrow). Enucleation was performed under anesthesia (arrowhead). **(b)** The right orbital socket healing is seen 3-, 19- and 57-days post-surgery (DPS). Exophthalmia (arrow) in the left eye have been noticed after 57 days post enucleation of the right eye. **(c)** Complete healing of right eye was observed 142 days post-surgery. Also, the healed surgical site is pigmented and the orbital socket has disappeared. Note to the progressive exophthalmia of the left eye 108- & 142-days post-surgery (DPS). **(d)** Necropsy performed 221-days post-surgery. Large mass (M) attached to the left testis was observed. Cut surface of the mass (M) was soft and whitish. Right testis (arrow) had normal appearance. Whitish nodule (arrowhead) is observed on the liver (L). Note the ovary (O) of hermaphrodite koi carp

In October 2020, a 2-year-old koi carp (*Cyprinus carpio*) was referred to the Ornamental Fish Clinic, Faculty of Veterinary Medicine, University of Tehran. The koi carp (*C. carpio*) was referred due to a large ocular mass with an ulcerated surface, extreme exophthalmia, and right eye hemorrhage (Fig. 1a). On clinical inspection, the mass was soft on palpation. Except for the extreme exophthalmia, no abnormal behavioral changes in the affected fish were observed. The mass had been observed one month prior to the submission and showed progressive growth. On gross examination, koi carp measured 25 cm and weighed 180 g in total body length and body weight, respectively. Although the affected fish had been kept with 4 other koi carp in the same aquarium, no mass had been observed in other tank mates. Wet mounts of skin, gills, and feces were prepared and observed by light microscopy (E600; Nikon). The fish were then anesthetized with the aquatic anesthetic “PI222” (100 ml/l; the main active ingredients of the PI222 are Eugenol, Carvacrol and Eugenol acetate) (Pars Imen Daru, Iran). PI222 was administered by immersing the animal in a PI222 solution. Enucleation was performed under anesthesia depending on the special condition and requirement of the fish for surgical intervention. Hemostasis was achieved using cautery. The orbital socket was left open to heal after surgery [31–33]. Tetracycline (5 mg/L) was added to the tank water postoperatively and was repeated on day 3 after a 50% water change. After the initial treatment, 50% of the tank water was changed on days 6 and 9 [34]. Following the enucleation, the wound and fish behavior were monitored until day 221. The overall condition of the fish improved after surgery, the process of skin formation began slowly (Fig. 1b), and complete healing of the right eye was observed 142-days post-surgery (DPS) (Fig. 1c). Exophthalmia in the left eye (Fig. 1b) has been noticed after 57 days post enucleation of the right eye. Despite antibiotic prescriptions, the exophthalmia of the left eye progressed (Fig. 1c). After 210 days, some clinical signs, including lethargy, anorexia, and imbalance in swimming, were observed. 211 DPS fish were found moribund. As a result of the poor prognosis and the owner’s consent, the fish was euthanized by an overdose of PI222. In the current study, the euthanasia procedures were in accordance with AVMA guidelines for animal euthanasia [35]. Necropsy was performed under sterile conditions. Aerobic and anaerobic bacterial cultures from the liver, kidney, and masses were incubated at 25 °C. For histological examinations, all masses and internal organs were dissected and fixed in 10% neutral buffered formalin before being dehydrated in ethanol series and embedded in paraffin with a paraffin tissue processor and paraffin dispenser. Several 5 μm sections were cut and stained with haematoxylin and eosin (H&E). In addition, immunohistochemical studies on mass sections were performed using primary antibodies against CD3 and CD20. The antibodies used were rabbit polyclonal anti-CD3 (T lymphocyte; Biocare) and rabbit polyclonal anti-CD20 (B lymphocyte; Thermo Fisher Scientific). Slides were counterstained with hematoxylin. The sections of the masses were scanned by the Plustek OpticLab H850 slide scanner. Also, sections were examined by light microscopy (E600; Nikon), and representative images were taken using an IDS UI-2250 microscope camera (IDS imaging).

No bacterial growth was observed on blood agar. Also, no external or internal parasites were revealed during microscopic examination of the internal organs, fins, gills, and skin scrapes. The fish was hermaphrodite, with both right and left testicles and one ovary. A large mass attached to the left testis and small whitish nodules on the surface of the liver were clearly visible (Fig. 1d).

**Fig. 2:**
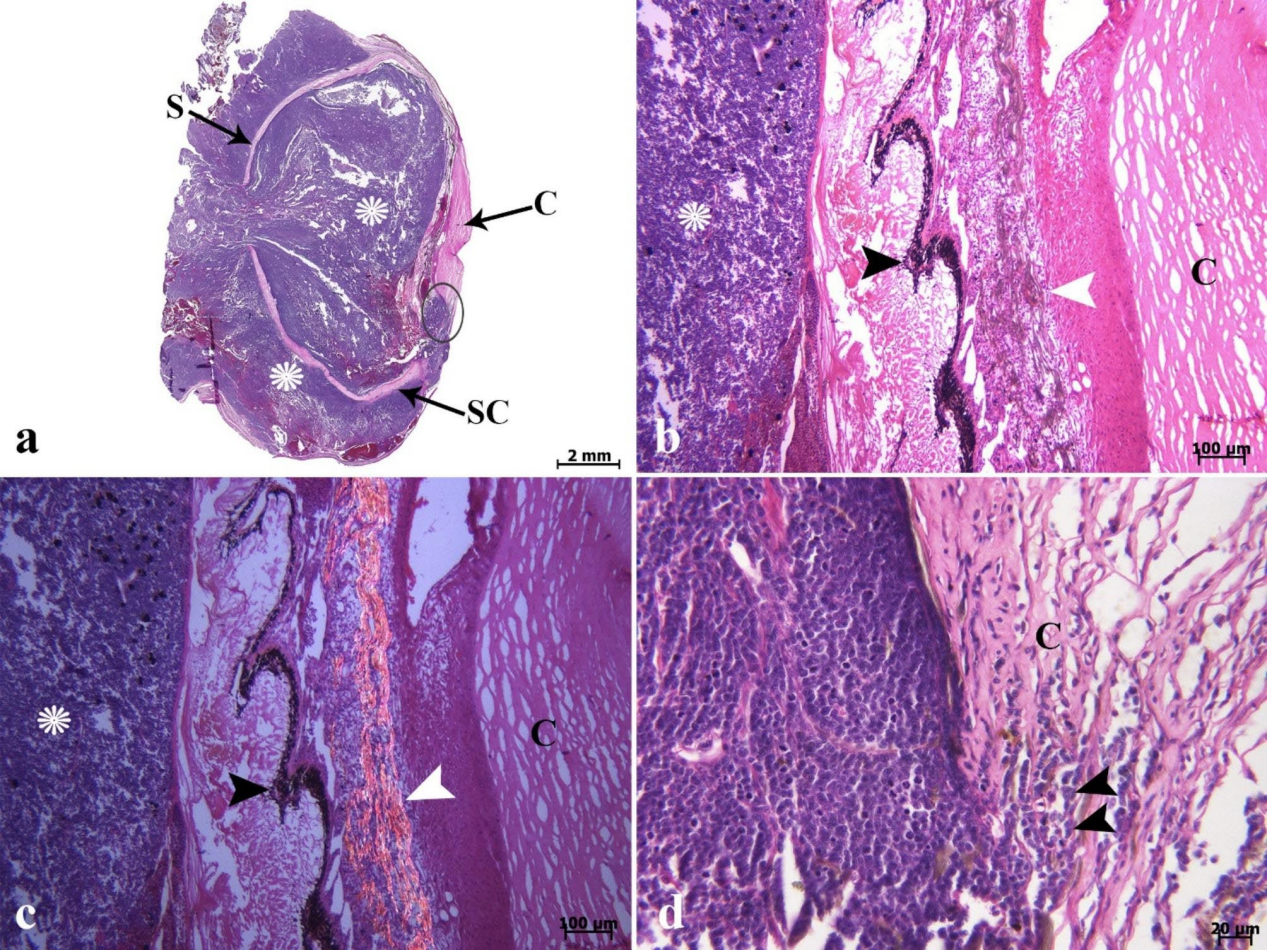
Lymphoma. Histopathological findings of the right ocular mass (**a-d**). **(a)** Scanned image of vertical cross-section of the right eye shows invasion of basophilic neoplastic cells (*) into (chamber of the vitreous body) and around the right eye from the outermost layer of posterior part of the eye (sclera) (S) until outermost layer of anterior portion (cornea) (C, ellipse). Scleral cartilage (SC). **(b & c)** Basophilic neoplastic cells (*) filled the chamber of the vitreous body until pigmented layer of iris. Iridophores (white arrowhead) are characterized at the light microscopic level by the presence of olive-green pigments which are birefringent with polarized light. Melanin (black arrowhead) and cornea (C). **(d)** Higher magnification (ellipse in Fig. 2a) shows invasion of neoplastic cells (arrowheads) to the corneal stroma (C) (H&E).

**Fig. 3:**
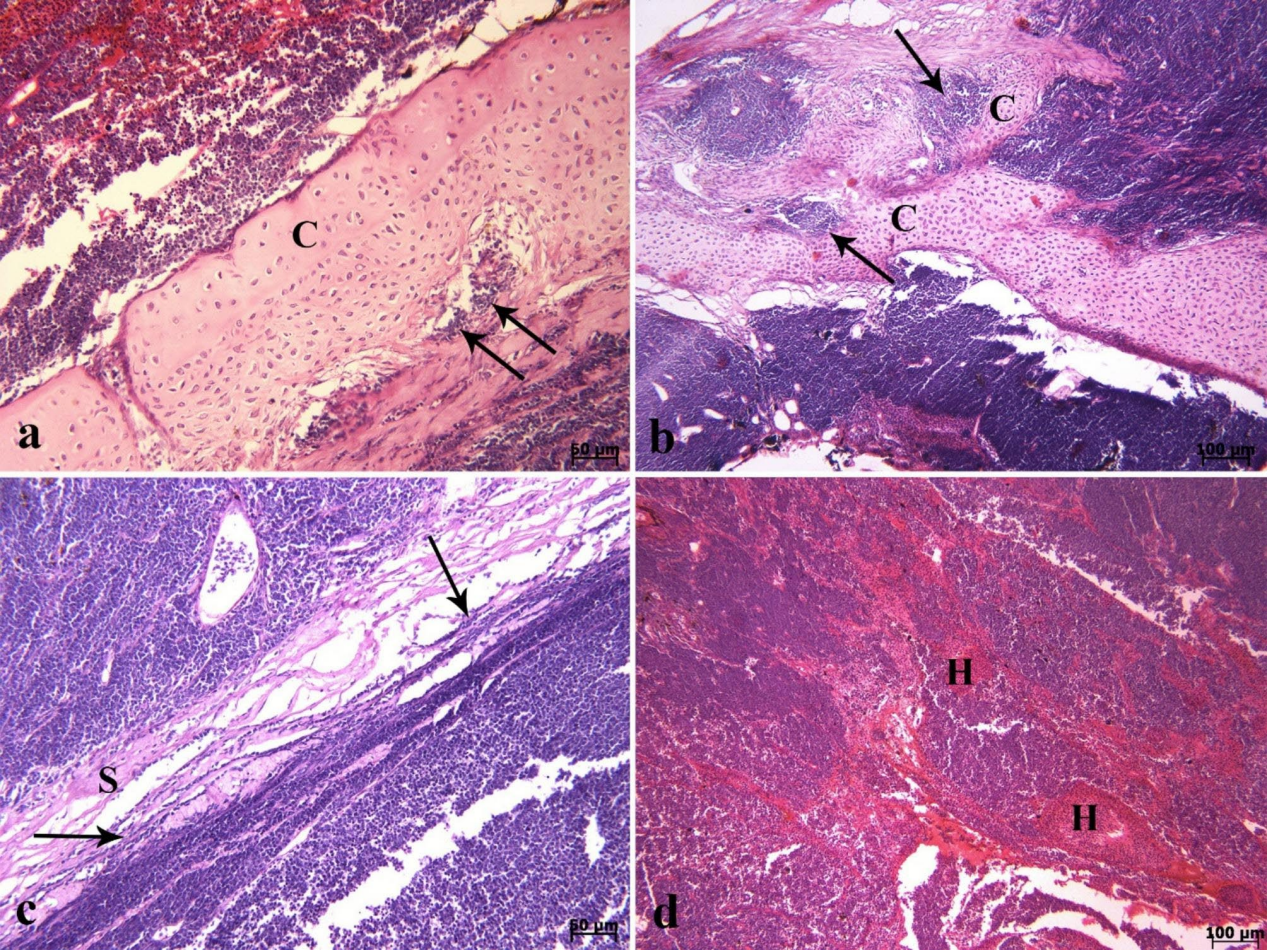
Lymphoma. Histopathological findings of the right ocular mass **(a-d)**. **(a & b)** Neoplastic cells invaded (arrows) to the different parts of scleral cartilage (C) (H & E). **(c)** Invasion of neoplastic cells (arrows) to the sclera (s) is seen. **(d)** Note to severe hemorrhage (H) in the eye mass (H & E).

**Fig. 4:**
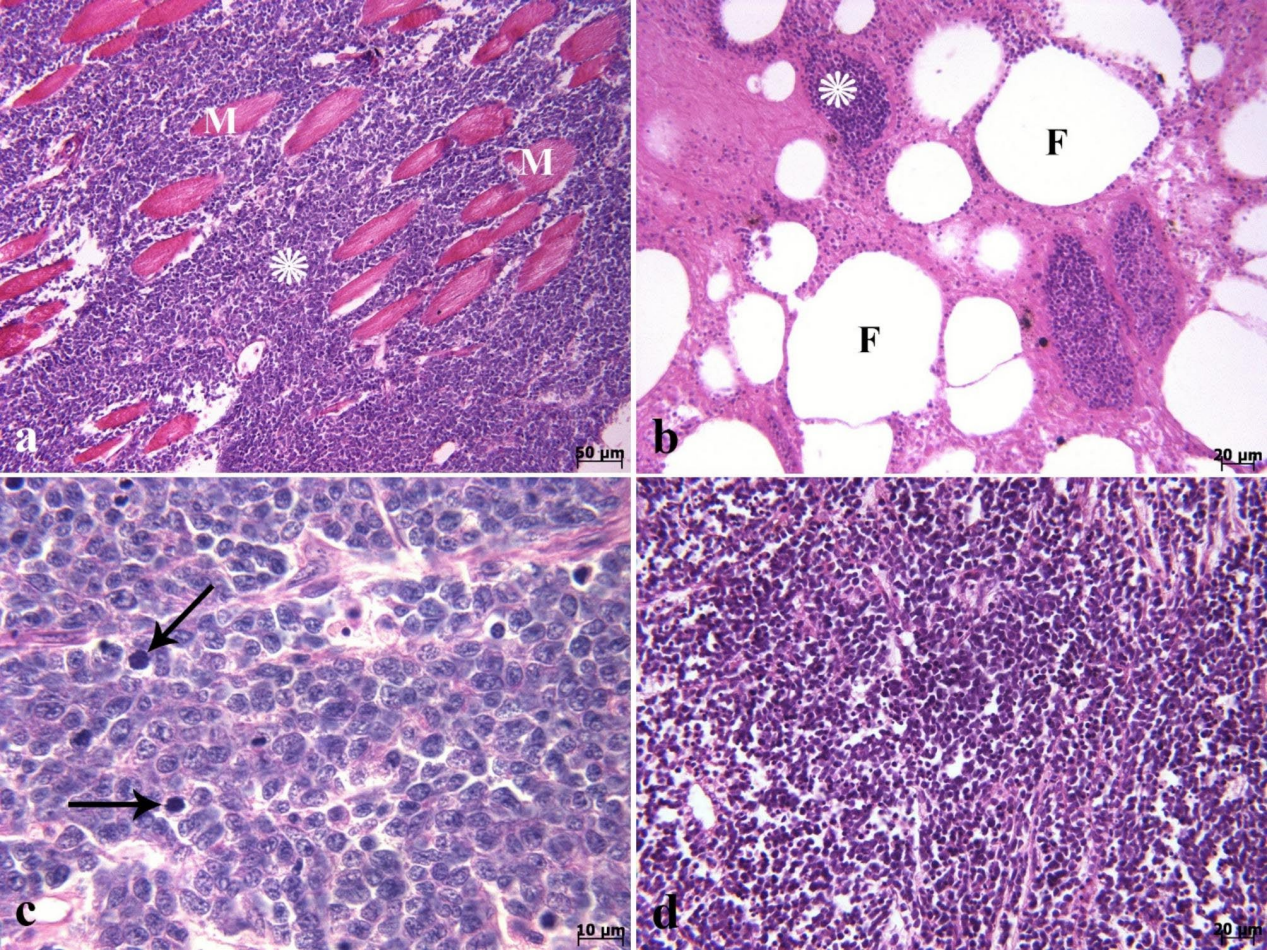
Lymphoma. Histopathological findings of the right and left ocular mass **(a-d)**. **(a & b)** Invasion basophilic neoplastic cells (*) between the extraocular muscle fibers (M) and adipose tissue (F) are seen. **(c)** Note the mitotic figures (arrows) in higher magnification. **(d)** Diffuse basophilic neoplastic cells are seen in the left eye (H&E).

**Fig. 5:**
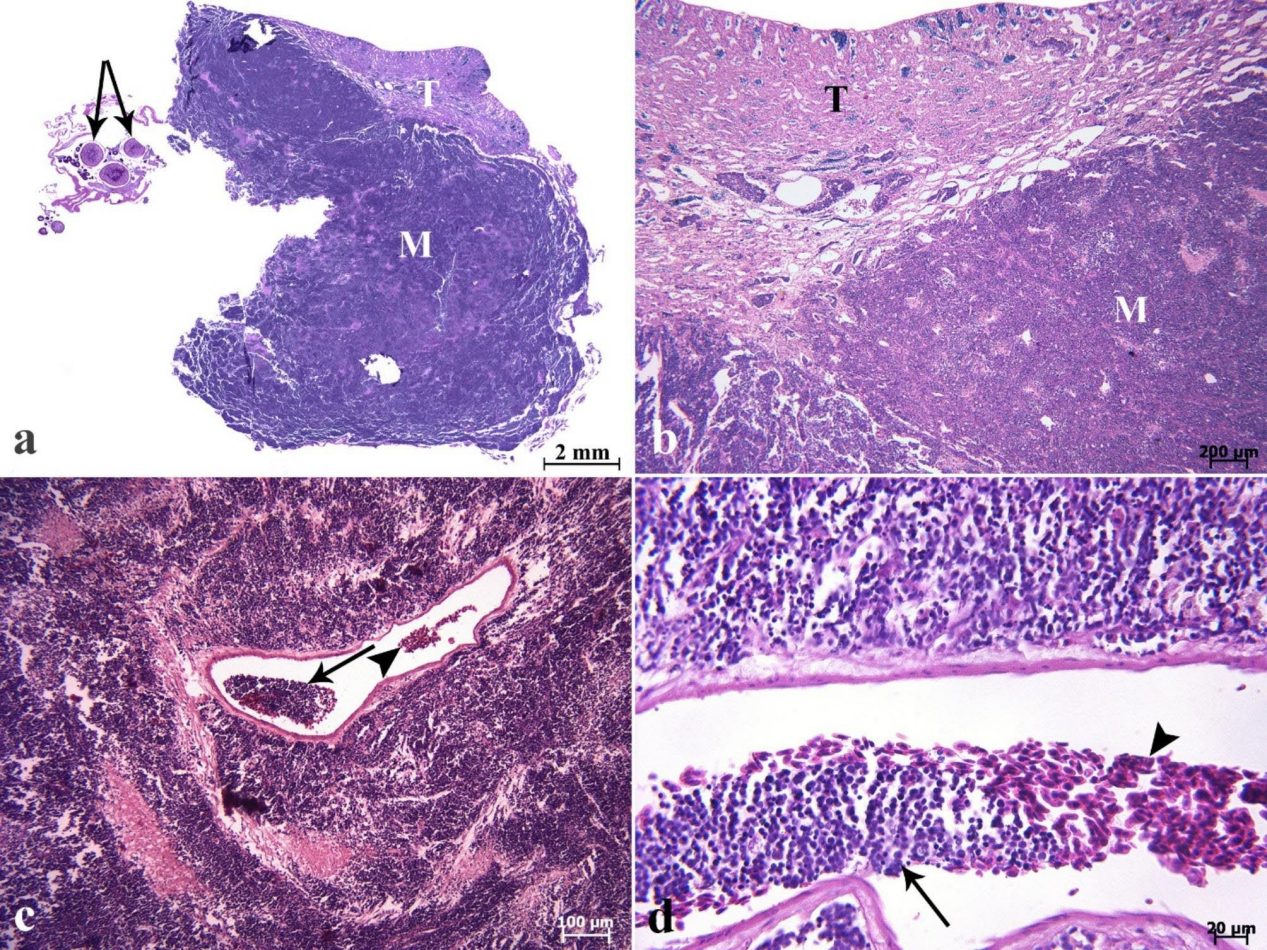
Lymphoma. Histopathological findings of testicular mass **(a-d)**. **(a)** Scanned image of cross-section of the testicular mass (M). Testicular tissue (T). A minor area of ovarian tissue associated with follicles (arrows) in ovotestis are seen. **(b)** The major part of left gonad (T) was occupied by the sheets and clusters of densely neoplastic lymphocytes of the mass (M). **(c)** Neoplastic cells (arrow) are seen in the vessel in the central part of the testicular mass. Red blood cells (arrowhead). **(d)** Higher magnification shows red blood cells (arrowhead) and neoplastic cells (arrow) in the vessel (H&E).

**Fig. 6:**
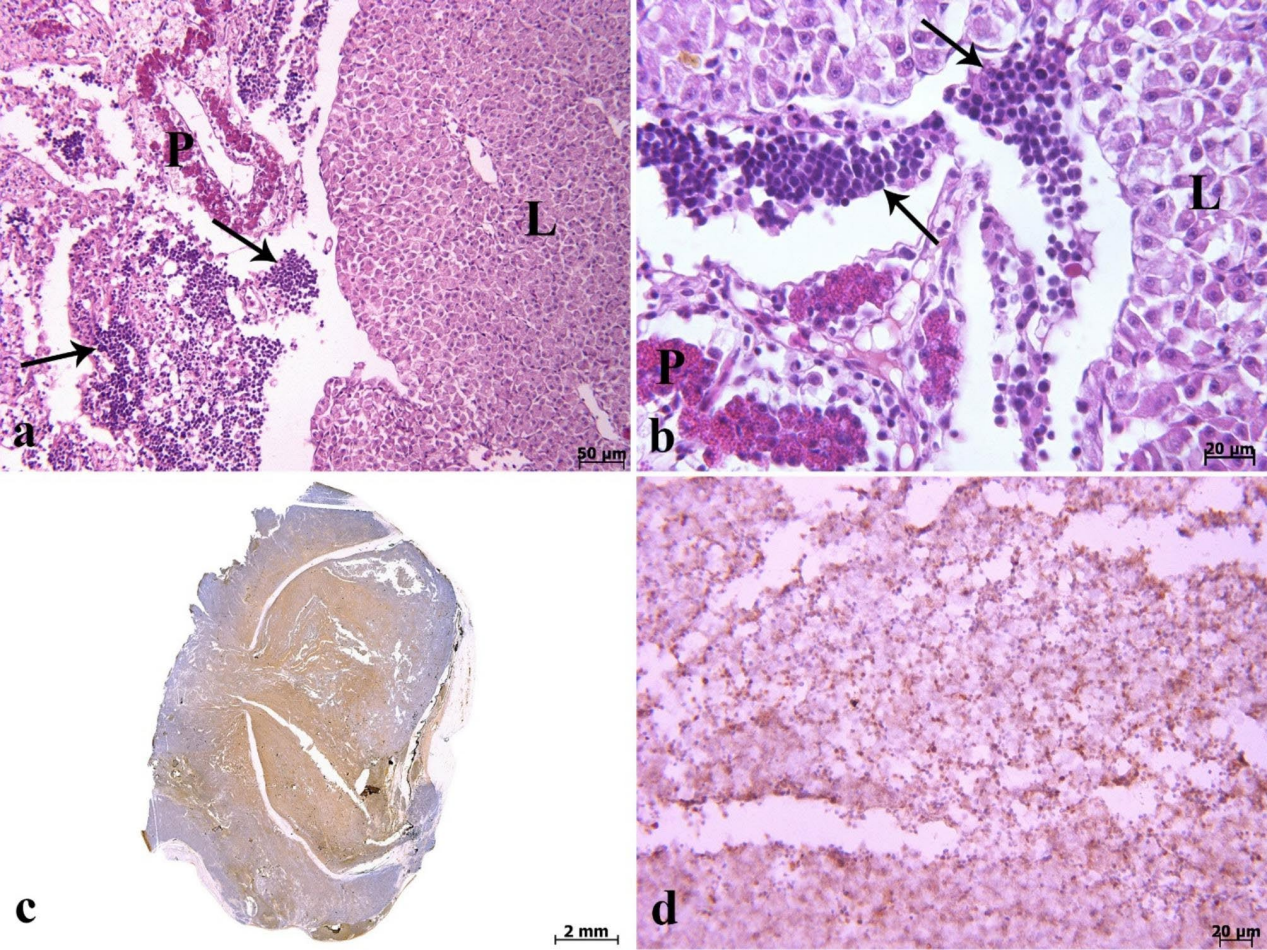
Histopathological & immunohistochemical staining of lymphoma. **(a & b)** Note to the metastasis of neoplastic cells (arrows) to the liver (L). Pancreas (P) (H&E). **(c)** Immunohistochemical staining of the right ocular mass for CD3 showing diffuse and strong positivity. **(d)** Positive staining of testicular mass with CD3.

On histopathological investigation, the right ocular mass was hypercellular in scant connective tissue. This mass was composed of sheets and clusters of densely and uniformly basophilic lymphoid cells involving the three tunics (the fibrous, vascular, and neuroepithelial), anterior and posterior chambers, extraocular muscles, and adipose tissue. These cells were extended from behind the bony orbit, through the extraocular muscle layer, adipose tissue, the eye globe, and onto the cornea (Fig. 2a-d). At higher magnifications, the corneal stroma (Fig. 2d), scleral cartilage (Fig. 3a & b), and sclera (Fig. 3c) were also infiltrated by neoplastic lymphocytes. In addition, multifocal hemorrhages (Fig. 3d), and invasion of basophilic neoplastic cells between the muscle fibers and adipose tissue (Fig. 4a &b) were observed in the sections. On microscopic examination, neoplastic cells were round to ovoid, bordered by a narrow rim of pale eosinophiliccytoplasm with an indistinct margin. The nuclei were round, with multiple nucleoli. Anisokaryosis and anisocytosis were mild to moderate. Mitotic figures (Fig. 4c) (mitotic count in 2.37 mm^2^) [36] were one to four per high-power field. The changes seen in the right eye indicated that an infiltrative, densely neoplastic cellular mass composed of round cells, similar to those seen in the left eye, was affecting the various parts of the eye (Fig. 4d). Histopathologically, samples of the left gonad showed testicular tissue and an area of ovarian tissue. In the ovarian component, there were some follicles. In the testicular component, the seminiferous tubules were also observed. Therefore, it was recognized as an ovotestis. The majority of the left gonad was occupied by sheets and clusters of densely neoplastic lymphocytes (Fig. 5a). These neoplastic cells had a small amount of eosinophilic cytoplasm, round nuclei, and multiple nucleoli, similar to those seen in the left and right eyes (Fig. 5b). Basophilic neoplastic cells were detected in blood vessels inside of the testicular mass (Fig. 5c & d), raising a suspicion of systemic spread. Microscopic metastasis, which had similar morphologic features as the ocular and testicular tumors, was observed in the liver (Fig. 6a & b). Despite the presence of neoplastic alterations in eye, gonad, and liver tissues, no histopathological changes were observed in the hematopoietic tissues of fish. Immunohistochemically, the neoplastic cells infiltrating the left and right eye, as well as the testicular mass, were positive for CD3 (Fig. 6c & d) but negative for CD20. T-cell lymphoma was diagnosed based on histopathological and immunohistochemical findings.

## Discussion and conclusions

Cancer is a multistep process and a disease of the genome, arising from DNA alterations that disrupt gene structure or function [37, 38]. The tumor classification system in veterinary oncology is based on the WHO (World Health Organization) histological classification of tumors of domestic species [39]. As in mammals, neoplasms of fish are clinicopathologically classified according to the histogenesis and the benign or malignant nature of the neoplasm [40, 41]. Lymphomas are the most common hematopoietic malignant neoplasms in humans and domestic animals [25, 28, 42]. In human oncology, lymphomas of T-cell origin are considered to be more aggressive and have a poorer prognosis in comparison with B-cell lymphoma [43, 44]. Furthermore, as in canines, the prevalence of T-cell lymphomas appears to be lower in fish than that of B-cell lymphomas. Generally, the thymus and kidney are described as the most common primary sites for neoplastic development [28, 45]. However, based on the location of lymphocytic cells in the early stages of the lesion, lymphomas may originate from other organs such as the testis and eye, justifying the diagnosis of oculo-testicular lymphoma in the current study.

The complementary diagnosis of lymphoma in the koi carp (*Cyprinus carpio*) was based on the light microscopic features and immunohistochemical characteristics of the neoplasm as described in both mammals and poikilotherms. Histopathologically, hypercellularity of mass in scant connective tissue was observed, which was consistent with the findings of Kieser et al. [24]. The majority of the neoplastic cells were round to ovoid and had eosinophilic cytoplasm with round nuclei and multiple nucleoli, which was in concurrence with the findings of Germann et al. [46]. The mass consisted of sheets of densely packed basophilic lymphoid cells, as described by Corapi et al. [47]. There was invasion of basophilic neoplastic lymphocytes into the corneal stroma, scleral cartilage, and sclera. Observation of several mitotic figures was consistent with the findings of Blazer and Schrank [25], Jung et al. [48], Corapi et al. [47], and Kasantikul et al. [28]. In addition, there was mild-to-moderate anisocytosis and anisokaryosis, which was in concurrence with the findings of Trope et al. [43]. Contrary to Thompson [21] and Bruno & Smail [26] observations, metastases in fish lymphoma have been reported previously. Liver metastasis of spontaneous stomach lymphoma in flowerhorn cichlids has been reported [27]. Also, metastasis in different organs in lymphoma in a Japanese medaka (*Oryzias latipes*) has been described [23]. The presence of similar morphologic features in the liver as the ocular and testicular masses confirmed the metastasis in the current study.

The presence of specific cytoplasmic immunolabelling for CD20 and CD79a suggested that neoplastic cells of lymphosarcoma in a captive bonnethead shark (*Sphyrna tiburo*) were most likely of B lymphocyte origin. Manire et al. [49] described the cross-reactivity of mammalian antibodies for B-lymphocyte markers CD79a and CD20 in the bonnethead shark (*S. tiburo*). Immunohistochemically, positive staining of the neoplastic cells infiltrating the left and right eye and testicular mass for CD3 and the negative staining for CD20 were consistent with the findings of Lakooraj et al. [50] and Namazi et al. [51]. These findings support the suggestion that immunohistochemical studies can be used as an additional method for diagnosing hematopoietic malignant neoplasms in some of fish species.

Retroviruses are well established as a cause of lymphoma in many domesticated mammal species [18] and are suspected of causing cutaneous lymphomas in pike and muskellunge [52, 53]. Although viral etiology is suspected in a number of fish hematopoietic tumours (e.g., lymphoma and lymphosarcoma) [30], there have been some reports of chemically-induced lymphosarcoma. Chen et al. [54] revealed that a chemical carcinogen such as N-methyl-N’-nitro-N-nitrosoguanidine plays a key role in the progression of lymphosarcoma in channel catfish (*Ictalurus punctatus*). Also, Schultz and Schultz [55] showed that 7, 12-dimethylbenz (a)-anthracene and diethylnitrosamine may be considered as a potential promoting factors for the development of lymphosarcoma in *Poeciliopsis*. Furthermore, Brown et al. [56, 57] established an association between the intensity of environmental contamination and the prevalence of lymphoma in pike (*Esox lucius*). Following observations on the ornamental fish farm, no more affected fish were discovered. In addition, no infectious agent was detected in the current study. Therefore, further research is needed to determine the cause of the lymphoid proliferation and the precise promoting factor for the development of this tumor in fish.

A final diagnosis of ocular and testicular T-cell lymphoma in the present study was based on the clinical signs, morphology, and texture of the tumour masses in the macroscopic and microscopic examinations. In addition, histopathological and immunohistochemical findings corresponded to T-cell lymphoma characteristics.

## Data Availability

The datasets used during the current study are available from the corresponding author on reasonable request.
